# Antiseptics in Railroad Surgery*Read before meeting of the Central Railroad Surgeons of Ga., at Columbus, April 20, 1892.

**Published:** 1892-05

**Authors:** C. H. Richardson

**Affiliations:** Montezuma, Ga.


### ANTISEPTICS IN RAILROAD SURGERY.*

BY C. H. RICHARDSON, M. D., MONTEZUMA, GA.

Antiseptic treatment of all wounds, from the smallest lacerated wound to the gravest operation, is now practiced by all enlightened and progressive surgeons. But in railroad surgery, antiseptic treatment is far more important, and the necessity far more urgent.

In this class of surgery we have not only to contend against the dirt and filth which they accumulate by their occupation, but also the general character of their wounds in their mangled and lacerated condition, require thorough cleanliness to get rid of the dirt—antiseptic treatment to prevent decay and death to the tissues.

There are no cases that come under the care of the surgeon that are so liable to undergo profuse suppuration and destruc-

*Read before meeting of the Central Railroad Surgeons of Ga., at Columbus, April 20, 1892.

tion of tissue as the lacerated wounds in railroad injuries. The physician who attempts to treat such injuries as he would other cases of accidents in civil life, without thoroughly cleansing the wound and pockets of all debris and foreign materials and dressing it under strict antiseptic precautions, is bound to meet with failures.

I have always made it a custom, when such cases are brought to my office, to first cleanse them in warm water and soap, and, after having gotten rid of the dirt and opened up all the pockets of clotted blood, to again cleanse it in bichloride of mercury solution 1-2000.

The following case, which was brought to my office in January, 1891, will illustrate more forcibly what I have to say:

Allen G., colored brakeman, who several days previously had received an injury in coupling cars. The hand was dressed by a physician (not a railroad surgeon) without any attention to cleanliness or antisepsis; in fact, I think it was dressed without even washing the blood off. The hand gave the most offensive odor, and gave evidence of a gangrenous condition. I undressed it and found considerable laceration of tissue on the index, middle and third fingers of the right hand, and the second and third phalanges of these fingers fractured and comminuted. I first cleansed it in carbolized water, and cutting off the devitalized tissue and skin, removing fragments of bone. Then thoroughly cleansed it again in bichlo. mercury solution 1-1000. There were numerous pockets where blood had undergone decay and filth from the discharges which had burrowed their way. These were all opened up and thoroughly cleansed. I placed the fingers in gutta-percha splints and dressed them with iodoform gauze, placing on top of this borated cotton to prevent constriction. On his return trip, in forty-eight hours, I again dressed it as before.

The hands are naturally the most unclean parts of the human system, and how often, in accidents to these members, they are brought to us when the hemorrhage has been checked by pitch and turpentine or cob-webs, or bundled up in old rags to keep, as they say, from "taking cold" in it.

All this requires thorough cleanliness, and cries aloud for antiseptic treatment. They should be thoroughly cleansed of foreign substances which have been used to check the hemorrhage, thoroughly irrigated with bichlo. of mercury solution 1-1000, all hemorrhage checked by catgut ligature, and the wounded surfaces dressed with bichlo. gauze. The old axiom, "cleanliness is next to godliness," is no less true to-day than in the time of our fathers. It not only gives health and life to lacerated tissues, but enables them to progress to a healthy condition and in much less time to recover. If this be true in minor operations, how much more important it is in all operations on a large scale.

In compound fractures we derive most gratifying results from antiseptic treatment. The old plan of converting compound fractures into simple ones by closing the wound with collodium is most unscientific. My plan has been when called to compound fractures of the leg, due to railroad injury, when the fleshy parts are necessarily contused and lacerated, is, as far as possible, to convert this lacerated wound into an incised one by cutting away much of the lacerated and devitalized tissue. If there be splinters of bone, these are taken out and sharp points of bone cut off. Then I wash the limb thoroughly in soap and water and ether; open all pockets of blood and thoroughly irrigate with bichlo. mercury solution. After having been satisfied that all dirt and foreign substances are removed, I put on an antiseptic bandage, and over this I apply the plaster Paris splint.

The following case came under my care in July, 1890:

Samuel Evans, colored, 46 years of age. On morning of 16th July, while handling cross-ties and throwing them, with the assistance of his fellow workmen, on the railroad car, his foot slipped and the whole tie came down on his leg, breaking it in the middle third. Not knowing his leg was broken, he jumped up and attempted to gather the tie again, when the bone shot out of the skin. I had him sent to a house, where I could conveniently look after him. When seen the upper fragment of tibia was projecting about 1 1-2 inches beyond the surface, and covered with dirt. I at once had his limb thoroughly cleansed with soap and water, and irrigated with bichlo. solution; put him under an anæsthetic, cut off the sharp pointed bone and placed it back in position ; cut off the ragged edges of tissue and brought the wound together, except about an inch left for drainage, with catgut ligature. I then irrigated again and wrapped the leg up in bichlo. gauze, and then put on the plaster of Paris splint, leaving a trap-door at the wound. This I allowed to stay on for four weeks without being disturbed. The most remarkable thing about this case was the small amount of discharge from this wound. I don't suppose the amount of pus, if it might be called pus, could have exceeded a teaspoonful. When the dressing over the wound became soiled, I redressed with iodoform gauze. At the end of four weeks I undressed it and reapplied the plaster Paris dressing as before.

In all amputations of whatever nature, from amputations of fingers to amputations at hip-joint, I generally render the parts to be amputated, the operator and all instruments, as aseptic as possible. I first shave the parts well and wash with soap, water and ether. I place under the patient, in capital operations, a rubber cloth wet with bichlo. solution to carry off the fluids. If it be a case of considerable laceration or crushing, I envelop the parts in towels wrung out in 1-2000 of bichlo. solution. To control hemorrhage in ordinary cases I use the rubber bandage by encircling the limb with it; tie the arteries with catgut ligatures and dress by sprinkling iodoform on the flaps and iodoform gauze as an outer dressing. When dressing becomes much soiled or offensive, I remove it and apply a fresh one, as before.

It seems to me that it is our imperative duty, when called to any case of railroad injury, to not only carry along all instruments and anæsthetics that may be needed to perform any operation, but also our bichlo. mercury tablets for making the solutions, solution for instruments, and several yards each of bichlo. and iodoform gauze. These can be easily carried in our satchel of instruments, and never should we attempt to dress a wound without dressing it antiseptically; for it often happens that these injuries occur off in some "out of the way" place, far from any drug store, and we must be prepared to treat at once, and treat as we would expect the treatment continued in order to give the best results, or as we would expect it to be the only treatment given, for it often happens in minor accidents, after dressing, this is the last seen of the patient, and if we desire a happy termination of the case, we should treat it upon the most scientific principles.

The field of railroad surgery is a broad one, and there will continue to be advancement in its scientific researches; but in no other field do we expect happier results than in the antiseptic treatment of wounds.

